# Discovery of a Novel *Parahenipavirus*, Parahenipavirus_GH, in Shrews in South Korea, 2022

**DOI:** 10.3390/v17060867

**Published:** 2025-06-19

**Authors:** Gyuri Sim, Chi-Hwan Choi, Minji Lee, Hak Seon Lee, Seong Yoon Kim, Seung Hun Lee, Hee Il Lee, Yoon-Seok Chung

**Affiliations:** 1 Division of High-Risk Pathogens, Department of Laboratory Diagnosis and Analysis, Korea Disease Control and Prevention Agency, Cheongju 28159, Republic of Korea; gyuris115@korea.kr (G.S.); chihwanchoi@korea.kr (C.-H.C.); mjlee4245@korea.kr (M.L.); lee5196@korea.kr (S.H.L.); 2 Division of Vectors and Parasitic Diseases, Department of Laboratory Diagnosis and Analysis, Korea Disease Control and Prevention Agency, Cheongju 28159, Republic of Korea; hslee8510@korea.kr (H.S.L.); gunbo0402@korea.kr (S.Y.K.); isak@korea.kr (H.I.L.)

**Keywords:** *Parahenipavirus*, shrew, zoonotic, *Henipavirus*, *Paramyxoviridae*

## Abstract

Highly pathogenic henipaviruses (Nipah and Hendra viruses) and parahenipaviruses (Langya virus) have demonstrated significant zoonotic potential. We aimed to identify *Henipavirus* or *Parahenipavirus* species in rodents and shrews in South Korea to underline the potential zoonotic transmission risk. Kidney and lung tissues from 285 rodents and shrews were screened for *Henipavirus* and *Parahenipavirus* using quantitative real-time reverse transcription polymerase chain reaction (qRT-PCR) targeting the Gamak virus and Daeryong virus (DARV) sequences. Based on the qRT-PCR results, 75 out of the 285 individuals tested positive, with the highest viral loads in the kidneys of *Apodemus agrarius*, *Crocidura lasiura*, and *Crocidura shantungensis*. A kidney sample from *C. shantungensis* that exhibited the lowest Ct value was further analyzed using PCR, Sanger sequencing, and metagenomic analysis, yielding a near-complete genome of a novel *Parahenipavirus*, designated Parahenipavirus_GH (PHNV-GH), which is phylogenetically related to DARV and Jingmen virus but exhibits distinct genomic features. *Ixodes granulatus* ticks were also identified on the host shrew. The identification of PHNV-GH in southern South Korea expands the known geographical distribution range of parahenipaviruses and highlights the ongoing risk of zoonotic transmission. Given the uncertain transmission dynamics and pathogenic potential of parahenipaviruses, comprehensive environmental surveillance and characterization of emerging parahenipaviruses are essential for preventing future outbreaks.

## 1. Introduction

Henipaviruses, members of the family *Paramyxoviridae*, are enveloped, negative-sense, single-stranded RNA viruses with genomes of approximately 18–19 kb, encoding six core proteins in the order 3′-N–P–M–F–G–L-5′ [1,2]. Recently, the International Committee on Taxonomy of Viruses (ICTV) established a new genus, *Parahenipavirus*, to distinguish rodent- and shrew-borne viruses from bat-borne viruses within the previously defined genus *Henipavirus* [3,4]. The new genus, *Parahenipavirus*, currently comprises 11 virus species, including *Parahenipavirus mojiangense* (Mojiang virus; MojV), *Parahenipavirus langyaense* (Langya virus; LayV), *Parahenipavirus jingmenense* (Jingmen virus), *Parahenipavirus gamakense* (Gamak virus; GAKV), and *Parahenipavirus daeryongense* (Daeryong virus; DARV) [4,5]. Henipaviruses exhibit an unusually broad host range. For example, fruit bats (*Pteropus* spp.) serve as natural reservoirs for Hendra virus (HeV) and Nipah virus (NiV), while several small mammals harbor newly recognized rodent- and shrew-borne parahenipaviruses (e.g., MojV, LayV, GAKV, and DARV), which can infect domestic animals and humans [5,6].

Notably, the 2001 and 2003 NiV outbreaks in Bangladesh, characterized by febrile neurological disease and human-to-human transmission, differed considerably from the earlier Malaysian outbreak [7]. This difference highlights the evolving nature of henipaviruses, which is further reinforced by the continued discovery of novel variants. Indeed, the discoveries of MojV in rodents in 2012 and Jeilong virus (JeiV) in bats in 2016 [8], followed by the LayV outbreak in China in 2022 [9], provide evidence of the persistent emergence and probability of the rapid spread of these potentially virulent pathogens.

**Figure 1:**
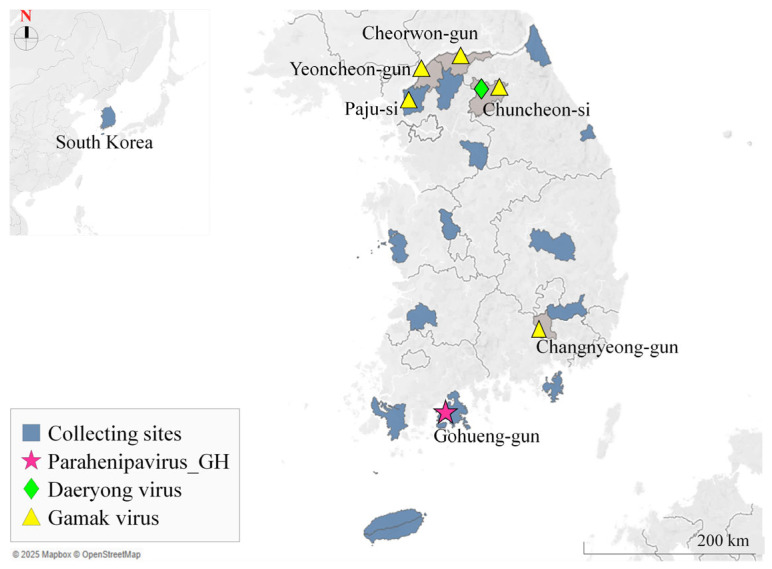
Map showing the present rodent- and shrew-sampling sites and the sites where parahenipaviruses were previously identified in South Korea. The sampling sites were Goheung-gun and Donghae-si in Gangwon-do; Paju-si, Pocheon-si, and Yeoju-si in Gyeonggi-do; Geoje-si in Gyeongsangnam-do; Cheongdo-gun and Uiseong-gun in Gyeongsangbuk-do; Goseong-gun and Haenam-gun in Jeollanam-do; Jeongeup-si in Jeonbuk-do; Jeju-si and Seogwipo-si in Jeju-do; Boryeong-si in Chungcheongnam-do; and Sejong-si. The blue areas on the map indicate the sampling sites in this study and the magenta star (★) represents the location where Parahenipavirus_GH was detected. The green diamond (◆) and yellow triangles (▲) indicate the sites where Daeryong virus and Gamak virus, respectively, were previously identified [11]. The map was generated using Tableau Public.

In South Korea, recent surveillance has contributed to the identification of novel paramyxoviruses, including Paju Apodemus paramyxoviruses (PAPVs) in *Apodemus agrarius* and GAKV and DARV in *Crocidura* species (Figure 1). Genetic analysis of these isolates has revealed considerable divergence from previously characterized viruses [8,9,10,11]. Although human infections have not been reported to date, the presence of these viruses in natural hosts, such as shrews and mice [10,11], necessitates continued surveillance to assess the potential risk of transmission.

Herein, we describe the identification of Parahenipavirus_GH (PHNV-GH), a previously unreported *Parahenipavirus*, in shrews captured in Goheung-gun, Jeollanam-do, South Korea. The findings in this study emphasize the need for sustained surveillance to identify animal hosts of *Parahenipavirus*, particularly shrews, and to assess their potential role as primary hosts for this virus.

## 2. Material and Methods

### 2.1. Sample Collection

Between October and November 2022, small mammals, including 285 rodents and shrews, were systematically collected across 15 regions in South Korea (Figure 1). Hard ticks were meticulously removed from the fur of animals using fine brushes, and the lung, kidney, and other tissue samples were obtained from these animals. Both tick and tissue samples were preserved at −80 °C for further analysis. All animal handling procedures in this study were approved by the Institutional Animal Care and Use Committee of the Korea Disease Control and Prevention Agency (Approval No. KDCA-IACUC-24-019), thereby ensuring adherence to strict ethical and scientific care guidelines.

### 2.2. Molecular Analysis

For molecular analysis, the lung and kidney tissue samples (10 mg) from the small mammals, along with the hard tick specimens, were individually homogenized using a Precellys Evolution homogenizer (Bertin Technologies, Montigny-le-Bretonneux, France). RNA was extracted from these homogenates using the KingFisher Flex System (Thermo Fisher Scientific, Waltham, MA, USA). To accurately identify the small mammals and hard ticks, we amplified cytochrome *b* (*Cytb*) and mitochondrial cytochrome *c* oxidase subunit I (*COI*) sequences, respectively (as described in the Supplementary Materials) [12,13].

We used the lung and kidney tissues from 285 rodents and shrews to screen for *Henipavirus* or *Parahenipavirus* species using quantitative real-time reverse transcription polymerase chain reaction (qRT-PCR) with primers and probes specifically designed for this study based on the previously reported sequences of GAKV and DARV [11]. The PCR mixture contained TaqPath 1-step RT-qPCR Mastermix (A15299; Applied Biosystems, Frederick, MD, USA) in a total reaction volume of 20 μL, including 5 μL of the extracted RNA. The primers and probes were synthesized using the custom TaqMan Gene Expression Assay by Thermo Fisher Scientific. Thermal cycling was conducted according to the manufacturer’s protocol: uracil-N-glycosylase activation at 25 °C for 2 min and reverse transcription at 50 °C for 15 min, followed by denaturation at 95 °C for 10 min, and 40 cycles of amplification at 95 °C for 3 s and 60 °C for 30 s. All reactions were performed on the QuantStudio™ Dx Real-Time PCR Instrument (REF 4470660; Applied Biosystems, Singapore). The threshold of qRT-PCR was set automatically, and the threshold cycle (C_t_) value was used to interpret the results (a detectable C_t_ value was used to define a positive result). After screening a total of 570 samples, an additional qRT-PCR analysis was performed on a single sample that exhibited the lowest C_t_ value, indicative of the highest viral load. For this analysis, RNA was extracted from the heart, liver, and spleen tissues, as well as the ectoparasitic ticks collected from the same host individual. Among the positive samples, several samples with relatively low C_t_ values were further analyzed for *Paramyxoviridae* subfamily members using established PCR protocols [14].

### 2.3. Sequence Characterization

To further characterize samples in which a *Paramyxoviridae*-associated target gene was amplified, we first synthesized complementary DNA (cDNA) from the extracted RNA and performed Sanger sequencing, followed by BLAST analysis (version 2.16.0). Subsequently, metagenomic analysis using whole-genome sequencing was conducted to comprehensively examine the genetic composition and diversity of the samples. cDNA libraries were prepared according to the manufacturer’s instructions using Illumina Viral Surveillance Panel 2 (VSP2; Illumina, San Diego, CA, USA), and the libraries were sequenced on an Illumina MiSeq platform with MiSeq Reagent Kit v2 (2 × 150 cycles). Raw reads were processed using fastp v0.23 to remove adapter sequences, low-quality reads (Q < 30), and short reads (<50 bp). Host-derived sequences were removed by mapping to the *Crocidura shantungensis* mitochondrial genome (GenBank accession no. NC_021398). The resulting high-quality paired-end reads were assembled de novo using SKESA v2.5.1 and the assembled contigs were used in the BLAST analysis.

The BLAST analysis of the contigs identified DARV (GenBank accession no. MZ574409) and Jingmen virus (GenBank accession no. OM030315) as the closest reference genomes. Accordingly, reference-based mapping was performed using MZ574409 in CLC Genomics Workbench v25.0.1 (CGW25) with default parameters. The final viral genome sequences were curated through manual inspection of both the de novo assembled contigs and reference-based mapping results. To complete the genome termini, unmapped and soft-clipped reads were analyzed, enabling the identification of the 3′ leader sequence (ACCAAACAAGGA) and 5′ trailer sequence (ACCACACAAGGA), which are structurally similar to conserved motifs found in other *Henipavirus* genomes. Although non-coding, these terminal sequences likely play key roles in replication and transcription regulation. Genome annotation was performed using CGW25 based on the finalized genome sequence.

### 2.4. Phylogenetic Analysis

For the phylogenetic analysis of viral genome sequences, trees were generated using the VipTree web server (version 4.0), which is used for whole-genome-based phylogenetic analysis of viruses [15]. Whole-genome sequences of PHNV-GH and recently described Parahenipaviruses, which are absent in the default VipTree database, were submitted in FASTA format. Pairwise proteomic similarity scores were computed via tBLASTx with default settings, transformed into evolutionary distances, and subsequently used to infer a neighbor-joining tree with the BIONJ algorithm [15].

The genome organization of PHNV-GH was compared with that of closely related henipaviruses and parahenipaviruses using CGW25. Reference sequences included the sequences of DARV, Jingmen virus, MojV, GAKV, LayV, Ghana virus, Cedar virus (CedV), NiV, and HeV.

## 3. Results

### 3.1. Mammalian Hosts of Parahenipavirus

On the bases of the molecular and sequence analyses of samples obtained from the 285 mammals, we identified *Apodemus agrarius* (striped field mouse, 69.1%), followed by *Crocidura lasiura* (Ussuri white-toothed shrew, 23.5%), *Crocidura shantungensis* (Asian lesser white-toothed shrew, 6.3%), and *Micromys minutus* (harvest mouse, 1.1%), as the predominant zoonotic hosts of *Parahenipavirus* (Table S1).

In the qRT-PCR analysis of the 570 tissue samples (kidney and lung) from the 285 small mammals, GAKV and DARV sequences were detected in 75 kidney samples (Table S1). Eight samples that exhibited the lowest C_t_ values—indicative of high viral loads—were from rodents (*A. agrarius*) and shrews (*C. lasiura*, and *C. shantungensis*) (Table 1). Positive qRT-PCR results were obtained exclusively for kidney samples, as summarized in Table S2; lung tissues yielded no detectable C_t_ values (not detected; N.D.). Notably, the individual that had the lowest C_t_ value for the kidney sample was identified as *C. shantungensis*, and all 10 ticks collected from this host were identified as *Ixodes granulatus*.

### 3.2. Identification of PHNV-GH

**Figure 2:**
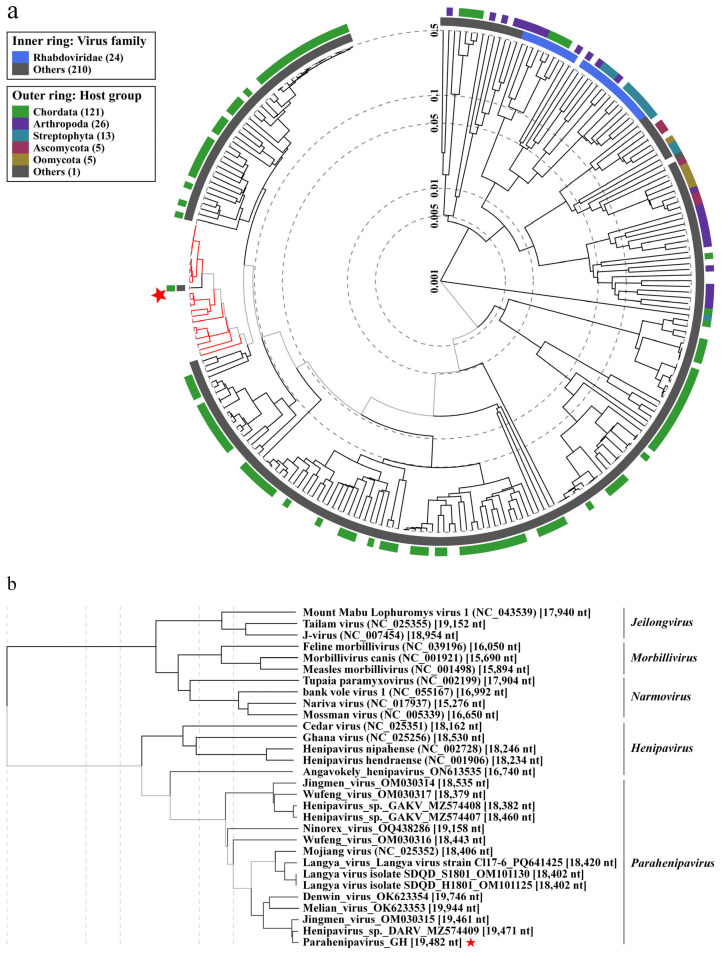
Phylogenetic analysis of a novel *Parahenipavirus*, Parahenipavirus_GH, using VipTree. Phylogenetic trees were constructed based on whole-genome sequences to examine the evolutionary relationships of Parahenipavirus_GH (PHNV-GH) with (**a**) related single-stranded RNA viral genomes and (**b**) closely related members of the family *Paramyxoviridae*. The red star indicates the PHNV-GH sequence identified in this study and the red branches denote input sequences, including PHNV-GH sequence, which were not available in the VipTree database at the time of analysis.

**Figure 3:**
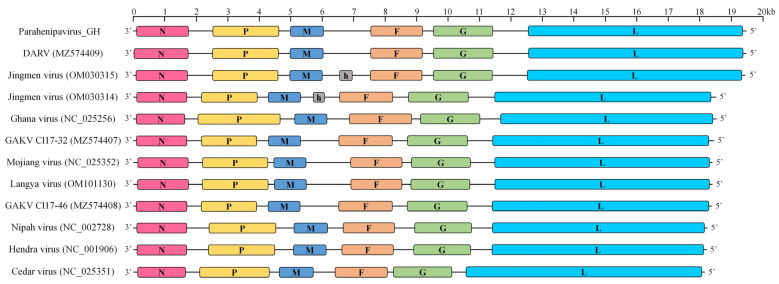
Genome organization of Parahenipavirus_GH and related *Henipavirus* and *Parahenipavirus* strains. This schematic diagram illustrates the genome organization of Parahenipavirus_GH compared with that of other reported henipaviruses and parahenipaviruses. The horizontal bars represent viral genomes, with colored segments indicating the coding regions of each gene. A scale bar indicating genome length is shown above the genome structures. Abbreviations: N, nucleocapsid protein; P, phosphoprotein; M, matrix protein; h, hypothetical protein; F, fusion protein; G, glycoprotein; L, large protein. The diagram was illustrated using Adobe Photoshop CS6.

Samples with lower C_t_ values were subjected to targeted PCR analysis based on previous *Paramyxoviridae* research [14], followed by Sanger sequencing and subsequent identification through NCBI BLAST analysis, confirming the presence of DARV-like sequences in one kidney sample of *C. shantungensis*. Metagenomic sequencing of the same sample using the Illumina MiSeq platform yielded a near-complete genome of a novel *Parahenipavirus*, designated PHNV-GH. To elucidate the evolutionary position of PHNV-GH within the genera *Henipavirus* and *Parahenipavirus*, we conducted phylogenetic analysis using VipTree, based on a comparison of full-length gene sequences. This analysis confirmed PHNV-GH as a member of the genus *Parahenipavirus*, revealing close relationships with DARV (GenBank accession no. MZ574409) and Jingmen virus (GenBank accession no. OM030315) (Figure 2). Average nucleotide identity (ANI) analysis between PHNV-GH and its two closest phylogenetic neighbors revealed 92.22% identity with DARV and 91.27% identity with Jingmen virus. Annotation of the open reading frames (ORFs) from 3′ to 5′ showed the genomic organization of nucleocapsid (N), phosphoprotein (P), matrix protein (M), fusion protein (F), glycoprotein (G), and large protein (L) (Figure 3). The genome of PHNV-GH is 19,482 nt in length, with its N-protein gene being 87 nt shorter than that of DARV. Collectively, the findings of these comparative genomic analyses provide convincing evidence for the classification of PHNV-GH as a novel species within the genus *Parahenipavirus*. The genomic sequences of PHNV-GH have been submitted to GenBank (GenBank accession no. PV712689).

### 3.3. Organotropism of PHNV-GH

Organotropism of PHNV-GH was investigated using qRT-PCR analysis, which revealed *Henipavirus* or *Parahenipavirus* genes in the kidney, liver, and spleen of the host shrew, but not in the heart or lung (Table 1). This distribution pattern suggests that PHNV-GH may exhibit organ-specific targeting. Notably, we failed to detect *Henipavirus* or *Parahenipavirus* RNA in any of the ticks collected from the same host shrew. This absence, which contrasts with observations regarding the known vectors of NiV and HeV, may contribute to the differences in organotropism identified in this virus [16].

## 4. Discussion

In this study, we performed qRT-PCR screening of 285 rodents and shrews and detected *Henipavirus*- or *Parahenipavirus*-like sequences in 75 individuals. In a kidney sample from *C. shantungensis* with the highest viral load, we identified a novel *Parahenipavirus*, designated as PHNV-GH. This shrew was captured in southern South Korea, and given that GAKV and DARV have been primarily detected in northern regions of the country, our findings broaden the known geographical distribution range of parahenipaviruses within South Korea (Figure 1).

Comparative analysis revealed that PHNV-GH shares genetic features and genomic organization with previously identified rodent- and shrew-borne parahenipaviruses, such as GAK, DARV, MojV, and LayV [11,17]. In contrast to CedV and MojV, which are currently considered non-pathogenic to humans, LayV has been associated with febrile illness in humans. During a LayV outbreak from 2018 to 2021, human infections occurred through unidentified transmission routes [9]. Notably, the identification of PHNV-GH in *C. shantungensis*, a shrew species known to be a host of LayV, raises important considerations regarding the zoonotic potential and reservoir competence of *C. shantungensis* [9,17].

Although no human infections with *Henipavirus* or *Parahenipavirus* have been reported in South Korea to date, several recently identified henipaviruses and parahenipaviruses, including CedV, MojV, and LayV, continue to be reported. The recurring detection of these viruses in wildlife strongly suggests a potential risk of zoonotic transmission [5,16]. In particular, habitat disruption driven by modern societal activities can increase the likelihood of human exposure to wildlife-borne pathogens, even in the absence of any direct animal contact, thereby heightening the risk for both human-to-human and animal-to-human transmission [11]. Consequently, comprehensive environmental surveillance to identify reservoir species and a thorough understanding of their ecological roles are essential for elucidating the mechanisms underlying viral transmission.

Although this study was conducted using archived samples and whole-genome sequencing was performed on a sample with the highest viral load to facilitate rapid identification of the novel virus, the detection of PHNV-GH highlights the importance of continued surveillance and molecular characterization of wildlife hosts, even in the absence of known human infections. The inference of PHNV-GH organotropism was based solely on qRT-PCR detection of viral RNA in available tissues, and further investigations are required to accurately confirm and analyze the actual organotropism of PHNV-GH.

Characterizing emerging henipaviruses or parahenipaviruses is essential for assessing the pathogenic potential and inter-species variability of these viruses, which will ultimately contribute to strengthening our ability to predict and prevent future zoonotic outbreaks.

## Figures and Tables

**Table 1:** qRT-PCR analysis for the detection of Gamak virus and Daeryong virus genes among multiple samples collected in South Korea.

Sample	Collection Area	Species	qRT-PCR C_t_ Values
Eight samples with the lowest C_t_ values			
#158 kidney	Jeollanam-do Goheung-gun	*Crocidura shantungensis*	24.209
#103 kidney	Gangwon-do Donghae-si	*Crocidura lasiura*	25.428
#243 kidney	Gyeonggi-do Paju-si	*Apodemus agrarius*	29.697
#244 kidney	Gyeonggi-do Paju-si	*Apodemus agrarius*	30.344
#160 kidney	Jeollanam-do Goheung-gun	*Apodemus agrarius*	30.797
#251 kidney	Gyeongsangbuk-do Cheongdo-gun	*Apodemus agrarius*	30.841
#240 kidney	Gyeonggi-do Paju-si	*Crocidura lasiura*	31.322
#189 kidney	Gyeongsangbuk-do Uiseong-gun	*Crocidura lasiura*	31.401
Other tissue samples analyzed from the individual with the lowest C_t_ value (#158)			
#158 lung	Jeollanam-do Goheung-gun	*Crocidura shantungensis*	N.D.
#158 heart			N.D.
#158 liver			32.22
#158 spleen			31.12

# indicates the sample number; N.D.: not detected.

## Data Availability

The genome sequence of Parahenipavirus_GH identified in this study has been deposited in GenBank under accession number PV712689. Other data generated and analyzed during this study are available from the corresponding author on request.

## Supplementary Materials

The following supporting information can be downloaded at: https://www.mdpi.com/article/10.3390/v17060867/s1, Supplementary Methods; Table S1: rodent and shrew specimens collected in South Korea in 2022, and qRT-PCR positivity for GAKV and DARV; Table S2: rodent and shrew samples with positive qRT-PCR results and Ct values.

## Abbreviations

The following abbreviations are used in this manuscript:

PCR	Polymerase chain reaction
qRT-PCR	Quantitative real-time reverse transcription polymerase chain reaction
GAKV	Gamak virus
DARV	Daeryong virus
MojV	Mojiang virus
LayV	Langya virus
PAPV	Paju Apodemus paramyxovirus
*Cytb*	Cytochrome *b*
*COI*	Cytochrome *c* oxidase subunit I
C_t_	Threshold cycle
CGW25	CLC Genomics Workbench v25
