# Supplementary material for: Discovery of a Novel Parahenipavirus, Parahenipavirus_GH, in Shrews in South Korea, 2022
Source: Viruses. 2025 Jun 19;17(6):867. doi: 10.3390/v17060867 (PMC12197346; doi:10.3390/v17060867)
Supplement: Supplementary file 1 [file viruses-17-00867-s001.zip › Table S1.pdf]

Supplementary Methods

Collection of small mammals and hard ticks

During October and November 2022, small mammals were collected using Sherman traps (3 × 3.5 × 9 inches; BioQuip, Gardena, California, USA) with peanut butter-spread biscuits at 15 regions in South Korea, namely, Pocheon, Donghae, Sejong, Boryeong, Cheongdo, Uiseong, Yeoju, Paju, Jeongup, Geoje, Goseong, Goheung, Haenam, Seogwipo and Jeju. The animal-handling protocol of this study was reviewed and approved according to the guidelines for ethical procedures and scientific care of the Institutional Animal Care and Use Committee of the Korea Diseases Control and Prevention Agency (KDCA-IACUC-24-019). The collected small mammals were euthanized using carbon dioxide and then dissected. To harvest parasitizing hard ticks, the bodies of the small mammals were suspended over bowls filled with tap water for 48 h. The hard ticks were collected from the surface using a fine brush. Lung and kidney tissues and hard ticks were frozen at -80°C until use.

Extraction of RNA and identification of small mammals and hard ticks

Ten milligrams each of lung and kidney tissues and hard tick samples was individually homogenized with a Precellys 2.8 mL Hard Tissue Reinforced ceramic Beads kit (Ck28-R) using a Precellys Evolution homogenizer (Bertin Technologies, Montigny-le-Bretonneux, France). RNA was extracted using the KingFisher Flex system (Thermo Fisher Scientific, Waltham, MA, USA) with the MagMAX™ *mir*Vanan Total RNA Isolation kit (Applied Biosystems, Waltham, MA, USA) according to the manufacturer's instructions. To identify the small mammals excluding *Apodemus agrarius*, which can be distinguished by its characteristic black stripe along the spine, cytochrome *b* (*Cytb*) was amplified using previously described oligonucleotide primers [9]. In addition, DNA primers designed by Folmer et al. [10] to amplify mitochondrial cytochrome *c* oxidase subunit I (*COI*) for diverse metazoan invertebrates were used for hard tick identification. Polymerase chain reaction (PCR) was performed with the AccuPower® PCR PreMix (Bioneer Inc., Daejeon, South Korea) in 20-μL reaction mixtures. All PCR products were sequenced in both directions using each PCR primer at Cosmogenetech (Seoul, South Korea).

Table S1. Rodent and Shrew Specimens Collected in South Korea in 2022 and qRT-PCR Positivity for GAKV and DARV

Species	Province (-do)	City (-si, -gun)	Number of samples	Positive for GAKV, DARV sequences (kidney samples)
Apodemus agrarius	Gangwon-do	Donghae-si	17	2/17 (11.8%)
		Goseong-gun	15	1/15 (6.7%)
	Gyeonggi-do	Yeoju-si	28	6/28 (21.4%)
		Pocheon-si	16	1/16 (6.3%)
		Paju-si	14	5/14 (35.7%)
	Gyeongsangnam-do	Geoje-si	2	0/2 (0%)
	Gyeongsangbuk-do	Cheongdo-gun	19	8/19 (42.1%)
		Uiseong-gun	6	1/6 (16.7%)
	Sejong-si	Sejong-si	24	3/24 (12.5%)
	Jeollanam-do	Haenam-gun	17	7/17 (41.2%)
		Goheung-gun	11	6/11 (54.5%)
	Jeonbuk-do	Jeongeup-si	13	3/13 (23.1%)
	Jeju-do	Seogwipo-si	7	2/7 (28.6%)
		Jeju-si	1	1/1 (100%)
	Chungcheongnam-do	Boryeong-si	7	3/7 (42.9%)
	Subtotal		197 (69.1%)	49
Crocidura lasiura	Gangwon-do	Goseong-gun	12	6/12 (50%)
		Donghae-si	6	1/6 (16.7%)
	Gyeonggi-do	Yeoju-si	6	1/6 (16.7%)
		Paju-si	5	2/5 (40%)
		Pocheon-si	3	0/3 (0%)
	Gyeongsangnam-do	Geoje-si	7	2/7 (28.6%)

	Gyeongsangbuk-do	Cheongdo-gun	5	1/5 (20%)
		Uiseong-gun	4	1/4 (25%)
	Sejong-si	Sejong-si	5	0/5 (0%)
	Jeollanam-do	Goheung-gun	4	0/4 (0%)
	Jeonbuk-do	Jeongeup-si	9	5/9 (55.6%)
	Chungcheongnam-do	Boryeong-si	1	0/1 (0%)
	Subtotal		67 (23.5%)	19
Crocidura shantungensis	Gyeongsangnam-do	Geoje-si	5	0/5 (0%)
	Gyeongsangbuk-do	Uiseong-gun	1	1/1 (100%)
	Sejong-si	Sejong-si	1	0/1 (0%)
	Jeollanam-do	Goheung-gun	3	3/3 (100%)
		Haenam-gun	3	1/3 (33.3%)
	Jeonbuk-do	Jeongeup-si	1	0/1 (0%)
	Jeju-do	Jeju-si	2	0/2 (0%)
		Seogwipo-si	1	0/1 (0%)
	Chungcheongnam-do	Boryeong-si	1	0/1 (0%)
	Subtotal		18 (6.3%)	5
Micromys minutus	Gyeonggi-do	Paju-si	1	0/1 (0%)
	Gyeongsangbuk-do	Uiseong-gun	1	1/1 (100%)
		Cheongdo-gun	1	1/1 (100%)
	Subtotal		3 (1.1%)	2
Total			285	75
